# Characteristics of Cerebrovascular Response to Intrinsic Vasoactive Substances in Sika Deer (*Cervus nippon yesoensis*) and the Possible Effects of Gravity on Adrenergic Responses

**DOI:** 10.3390/ani14233500

**Published:** 2024-12-04

**Authors:** Md. Zahorul Islam, Siyuan Wu, Tomoki Ootawa, Henry Smith, Ha Thi Thanh Nguyen, Etsumori Harada, Atsushi Miyamoto

**Affiliations:** 1 Department of Veterinary Pharmacology, Joint Faculty of Veterinary Medicine, Kagoshima University, 1-21-24 Korimoto, Kagoshima 890-0065, Japan; khokonpharma@gmail.com; 2 Department of Pharmacology, Faculty of Veterinary Science, Bangladesh Agricultural University, Mymensingh 2202, Bangladesh; 3 Department of Basic Veterinary Science, Joint Graduate School of Veterinary Medicine, Kagoshima University, 1-21-24 Korimoto, Kagoshima 890-0065, Japan; k0460053@kadai.jp (S.W.); k2773273@kadai.jp (T.O.); k5908476@kadai.jp (H.S.); 4 Department of Veterinary Pharmacology and Toxicology, Faculty of Veterinary Medicine, Vietnam National University of Agriculture, Gia Lam, Hanoi 131000, Vietnam; nguyenhavet@vnua.edu.vn; 5 Department of Veterinary Physiology, Faculty of Agriculture, Tottori University, 4-101 Minami, Koyama-cho, Tottori 680-8553, Japan

**Keywords:** acetylcholine, basilar artery, deer, gravity, noradrenaline, histamine

## Abstract

Gravity may exert species-specific effects on quadrupedal vasoreactivity, reflecting variations in the vertical displacement of the cardiocranial axis from the dorsal plane. Deer show markedly displaced cardiocranial axes compared to their closest phylogenetic relatives, but their relative cerebrovascular responses remain unelucidated. Accordingly, we investigated the responses to noradrenaline (NA), acetylcholine (ACh), 5-hydroxytryptamine (5-HT), histamine, angiotensin (Ang) II, and bradykinin (BK) in cervine basilar arterial rings. NA and 5-HT induced slight contraction, and ACh induced relaxation, which contrasts with the findings reported in pigs and cattle. The cumulative response to ACh was abolished by endothelial denudation and inhibited by *N^ω^*-nitro-L-arginine (a nitric oxide synthase inhibitor), atropine (a nonselective muscarinic antagonist), and p-fluoro-hexahydro-sila-difenidol (an M_3_ antagonist). Pirenzepine (an M_1_ antagonist) and methoctramine (an M_2_ antagonist) showed no significant effects. Histamine induced contractions, with its concentration–response curve shifted to the right in parallel by diphenhydramine (an H_1_ antagonist). However, cimetidine (an H_2_ antagonist) showed no significant effects. Ang II and BK had no vasomotive effects. NA and ACh induced different cerebrovascular responses in sika deer versus cattle, but histamine and BK did not. Our findings suggest that cerebrovascular responses are influenced by the similarity of animal species and the head and heart positions relative to gravity.

## Simple Summary

Animal species show marked variations in their cerebrovascular responses to substances that affect blood vessel tone, like noradrenaline, histamine, acetylcholine, 5-hydroxytryptamine, angiotensin II, and bradykinin. These variations may result from the receptor subtype distribution in smooth muscle or endothelial cells, and reflect the interplay of evolutionary and genetic influences for different animal species. Recently, we have investigated whether and how the response to one of these substances, noradrenaline, is related to deviations from an even heart-to-head alignment in an animal’s normal posture (which has implications for the gravitational effect on blood flow), for multiple species. Sika deer are even-toed ungulates, closely related to cattle. Close phylogenetic relatives have often been found to show similar cerebrovascular responses, but deer and cattle have a very different heart-to-head alignment. Accordingly, we compared the cerebrovascular responses between sika deer and other ungulates. We found that deer and other even-toed ungulates have similar cerebrovascular responses to histamine, bradykinin, *N^ω^*-nitro-L-arginine, and indomethacin (evaluated with cattle), but different responses to noradrenaline and acetylcholine (evaluated with pigs and cattle). We considered that cerebrovascular responses to noradrenaline may positively correlate with gravity (as it affects blood flow in a normal posture) in quadrupedal animals.

## 1. Introduction

**Figure 1:**
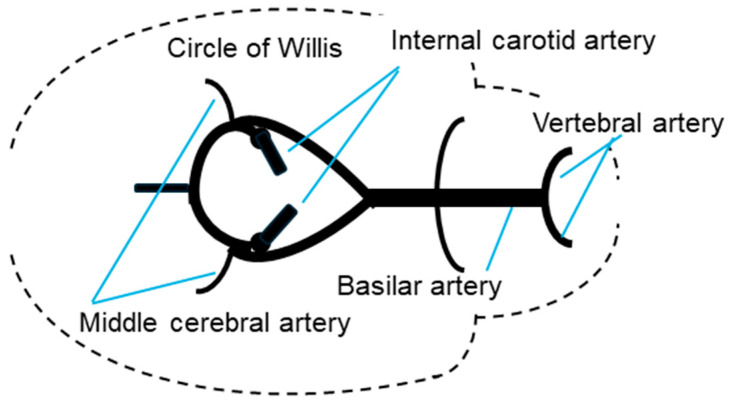
Main arteries of the cerebral base in deer. Dorsal view.

The cerebral vasculature has to maintain a stable blood flow to the brain despite changes in blood pressure. In part, this functionality relies on the Circle of Willis, where blood supplied from the heart to the brain enters the cerebral circulation, and blood vessels encompassed by this anatomical formation, including the basilar and middle cerebral arteries (Figure 1). The responses of these blood vessels are important in maintaining blood flow, with autoregulation, neurovascular coupling, endothelium-dependent responses, and vascular reactivity being especially important [1,2]. Cerebral blood flow is strictly regulated to ensure an adequate supply of oxygen to the brain. The reactivity of the cerebral artery plays a key role in cerebral autoregulation and determining local microvascular pressure. Changes in cerebral blood flow are controlled by alterations in the tone of vascular smooth muscle, which is directly or indirectly influenced by a range of endogenous vasodilators and vasoconstrictors [3]. These endogenous substances are activated or inactivated in response to stimuli including changes in shear stress [4], the partial pressure of arterial oxygen, arterial oxygen saturation, the partial pressure of carbon dioxide, and the pH [5,6].

Cerebrovascular responses to intrinsic vasoactive substances such as noradrenaline (NA), histamine, acetylcholine (ACh), 5-hydroxytryptamine (5-HT), angiotensin (Ang) II, and bradykinin (BK) have been investigated in various species, and animal species differences in response have been reported. For example, the basilar arteries from guinea pigs [7], humans [8], mice [9], rabbits [10], horses, and cattle [11] responded to histamine with contractions in the resting vascular tone, whereas the basilar arteries from bats [12], rats [7] and monkeys [13,14] responded with relaxation under the precontracted conditions. ACh induced relaxation in the basilar arteries of mice, bats, dolphins, and horses [9,12,15,16,17] but contraction in the basilar arteries in pigs and dogs [16,18], and it did not alter the tone of the bovine basilar artery [16,19]. Such intraspecific variation in vasoreactivity may be driven by factors such as receptor subtype distribution in smooth muscle or endothelial cells, and reflect the interplay of the evolutionary and genetic influences for different animal species.

Sika deer (*Cervus nippon yesoensis*) are even-toed ungulates (Artiodactyla) and wild ruminants that are prone to many of the same infectious diseases as cattle; however, their cerebrovascular responsiveness has not yet been studied. Most studies on the cerebrovascular responses in ruminants have involved the evaluation of domesticated animals. The morphology of cervid cerebral arteries is similar to that of other ruminants [20]. Information regarding cerebrovascular responsiveness may be useful during medical and surgical interventions. Deer are closely phylogenetically related to cattle [21]. Close phylogenetic relatives have often been found to show similar cerebrovascular responses; for example, the responses in dolphins resemble those in pigs, and the responses in bats resemble those in horses [12,15]. We have previously conducted comparative studies on basilar arterial vascular responsiveness in a wide variety of animals such as horse, cattle, pigs, dolphins, bats, and mice to determine their evolutionary linkages [9,12,15,16,22,23], testing hypotheses that the basilar artery would respond to more vasoactive substances as the brain evolves. More recently, we have focused on the relationship between the vertical displacement of the cardiocranial axis from the dorsal plane in an animal’s normal posture and NA-induced response in the basilar artery. In a resting vascular tone, NA constricts the basilar arteries in dogs [24], horses [17] and humans [25], relaxes the basilar arteries in cattle [26] and pigs [27], and does not affect the basilar arteries in mice [9] or rats [28]. In deer, the cardiocranial axis shows a similar displacement to that noted in dogs and horses, although deer are even-toed ungulates like pigs and cattle. Accordingly, we investigated the cerebrovascular responsiveness of sika deer to NA, histamine, ACh, 5-HT, Ang II, and BK to elucidate any differences between the responses in dogs, horses, pigs, and cattle, and to determine the correlation between the responsiveness to NA and the gravity in quadrupeds.

## 2. Materials and Methods

### 2.1. Tissue Preparation of Deer

Basilar arteries were obtained from the carcasses of Sika deer (*Cervus nippon yesoensis*, total number: 25, unknown age, both sexes, body weight of 10–34 kg) that were humanely slaughtered by electrical stunning and then the severance of their jugular vein, on a dedicated farm in Hokkaido, Japan, in accordance with the Japanese laws on the slaughter of animals for food consumption. Around 30 min were required to remove the blood vessels after euthanasia. The basilar artery was gently removed from the brain in each carcass, and transferred to ice-cold physiological saline (119 mmol/L NaCl, 4.7 mmol/L KCl, 1.6 mmol/L CaCl_2_, 1.2 mmol/L MgCl_2_, 25 mmol/L NaHCO_3_, 1.2 mmol/L KH_2_PO_4_, and 10 mmol/L glucose), pH 7.4, aerated with carbogen [95% (*v*/*v*) O_2_, 5% (*v*/*v*) CO_2_], and shipped to our laboratory. Each artery was then dissected and freed of adherent tissues using a stereomicroscope (Nikon, Tokyo, Japan). All the experiments were performed in accordance with the Kagoshima University Guidelines for Animal Experimentation. However, because this study involved arteries isolated from animals that had already been slaughtered for food consumption, it did not require ethical approval as an animal experiment. The animal experimental facilities and programs of the Joint Faculty of Veterinary Medicine, Kagoshima University have been fully accredited by AAALAC International since 2017.

### 2.2. Reagents

We used the following reagents at their final concentrations shown: NA (adrenergic receptor agonist, 10^−9^–10^−5^ mol/L, Tokyo Chemical Industry, Tokyo, Japan), histamine hydrochloride (histamine receptor agonist, 10^−8^–10^−3^ mol/L), diphenhydramine hydrochloride (H_1_ receptor antagonist, 10^−8^–10^−6^ mol/L, Sigma-Aldrich, St. Louis, MO, USA), cimetidine (H_2_ receptor antagonist, 10^−6^ mol/L, Sigma-Aldrich), methoctramine hydrate (M_2_ receptor antagonist, 10^−6^ mol/L, Sigma-Aldrich), atropine sulfate (nonselective muscarinic receptor antagonist, 10^−7^–10^−5^ mol/L, Sigma-Aldrich), Ang II acetate salt (angiotensin II receptor agonist, 10^−9^–10^−5^ mol/L), BK acetate salt (BK receptor agonist, 10^−10^–10^−6^ mol/L, Sigma-Aldrich), *N^ω^*-nitro-L-arginine (NO synthase inhibitor, L-NNA; 10^−4^ mol/L, Sigma-Aldrich), and sodium nitroprusside (NO donor, SNP; 10^−4^ mol/L) (Sigma–Aldrich). Indomethacin (cyclooxygenase inhibitor, 10^−5^ mol/L; Nacalai Tesque, Kyoto, Japan), 5-HT (serotonin)-creatinine sulfate (5-HT receptor agonist, 10^−9^–10^−5^ mol/L; Merck, Darmstadt, Germany), ACh chloride (ACh receptor agonist, 10^−9^–10^−5^ mol/L; Daiichi Sankyo, Tokyo, Japan), pirenzepine dihydrochloride (M_1_ receptor antagonist, 10^−6^ mol/L; Santa Cruz Biotechnology, Santa Cruz, CA, USA), *p*-fluoro-hexahydro-sila-difenidol hydrochloride (pFHHSiD, M_3_ receptor antagonist, 10^−7^–10^−6^ mol/L; Research Biochemicals, Natick, MA, USA), and U-46619 (thromboxane A_2_ analog, 10^−7^ mol/L; Cayman Chemical Company, Ann Arbor, MI, USA) were used. All the drugs were dissolved in distilled water.

### 2.3. Isometric Myography Studies

Four rings of approximately 4 mm in length were cut from each basilar artery. Each ring was horizontally mounted between two L-shaped stainless-steel holders (outer diameter, 0.1 mm), with one part fixed to an isometric force transducer (TB-611T, Nihon Kohden Kogyo, Tokyo, Japan), and immersed in a 4 mL water-jacketed micro tissue organ bath (UMTB-1, Unique Medical Co., Ltd., Tokyo, Japan) containing oxygenated salt solution at 37 °C (pH 7.4). Each suspended ring was allowed to equilibrate for at least 120 min under a resting tension of 0.75 g. This tension setting was chosen to allow the induction of maximum contractions in the basilar artery. KCl (60 mmol/L) was applied every 30 min until the amplitude of the contraction reached a constant value. Changes in the KCl concentrations of physiological saline were compensated for by adjusting with an equimolar concentration of NaCl. The isometric tension was recorded using an amplifier (AP-621G; Nihon Kohden Kogyo, Tokyo, Japan) and digitized using an analog-digital converter (Powerab/8SP; ADInstruments Co., Castle Hill, NSW, Australia), and the result was stored on the hard disk of a personal computer. The cumulative concentration–response curve for each agonist was obtained by directly adding a solution of the agonist to the fluid in bath. The contractile response was measured under a normal arterial resting tone, and the relaxation response was measured under contraction with U-46619 (10^−7^ mol/L; an analog of thromboxane A_2_). The contractile response was calculated as the percentage against the contraction at 60 mmol/L KCl, while the relaxant response was calculated as the percentage against the relaxation induced by sodium nitroprusside (10^−4^ mol/L). The response of a specific agent in the absence of an antagonist of that particular agent is considered the control. Antagonists were added to the bathing medium 30 min before adding the agonist. The antagonists did not affect the resting vascular tone. The log concentration ratio of the concentration producing a half-maximum response (EC_50_) in the absence or presence of an antagonist was calculated and plotted against the logarithm of the antagonist concentration to obtain the pA_2_ value [29]. Experiments were performed with a small number of samples (n = 25), as deer are wild and exotic animals, and obtaining samples is difficult.

### 2.4. Measurement of the Vertical Displacement of the Cardiocranial Axis of Deer, Horses, Mice, Cattle, Dolphin and Pigs

Triplicate photographs of deer, horses (Percheron-Breton-Belgian crossbreed), mice (4 months ± 15 days, ICR), cattle (about 2.5 years old, Japanese black beef oxen), dolphin (indeterminate age range, bottlenose), and pigs (6–7 months old, LWD crossbreed) were imported into a personal computer, and the angle between a dorsal plane passing through the base of the heart and the cardiocranial axis (defined as straight line connecting the base of the heart and center of the basilar artery) was measured using a web-based protractor, and the average of each was calculated. The anterior margin of the heart was approximately aligned with the third rib [30].

### 2.5. Statistical Analysis

The results were expressed as the means ± standard error of mean (SEM). Statistical analyses were performed using Student’s *t*-test after *F*-test or the Bonferroni test after a one-way analysis of variance after the Bartlett test (Stat View J-4.5; Abacus Concepts Inc., Berkeley, CA, USA). The results were expressed as the mean. Peason’s correlation test was applied to evaluate the relationship between the response to noradrenaline and the vertical displacement of the cardiocranial axis in quadrupedal animals (Excel 2016, Microsoft, Redmond, WA, USA). A statistical significance was established at *p* values < 0.05.

## 3. Results

### 3.1. Responsiveness to NA, ACh, 5-HT, Histamine, Ang II and BK

**Figure 2:**
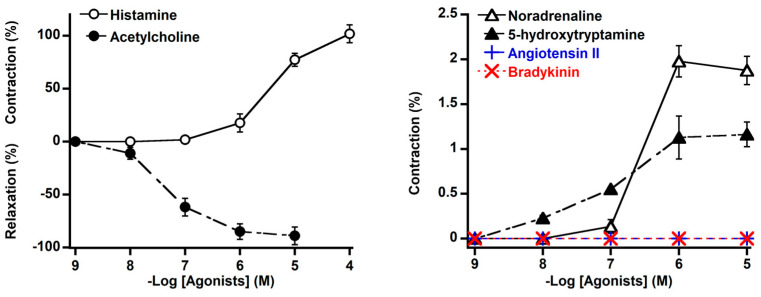
Effect of histamine (○), acetylcholine (●), noradrenaline (△), 5−hydroxytryptamine (▲), angiotensin II (+), bradykinin (×), and on isolated basilar arteries of deer. Contraction response was measured under resting tension and calculated as percent response to 60 mmol/L KCl, and relaxation in response to acetylcholine was assessed in the arteries precontracted with U-46619 (10^−7^ mol/L) and calculated as percent response to 10^−4^ mol/L sodium nitroprusside. Each point represents mean ± SEM of five deer.

We generated concentration–response curves for NA, ACh, 5-HT, histamine, Ang II, and BK using the isolated basilar arteries. NA and 5-HT induced very a weak contraction (1–2% vs. 60 mmol/L KCl). Histamine induced a contraction, whereas ACh induced relaxation in a concentration-dependent manner. Ang II and BK did not induce vasomotor activity (Figure 2). The maximum contractile responses (% vs. 60 mmol/L KCl) and relaxation responses (% vs. response to 10^−4^ mol/L SNP) to these vasoactive substances are shown in Table 1.

### 3.2. Responsiveness to L-NNA and Indomethacin Under Resting Tension

L-NNA (an NO synthase inhibitor, 10^−4^ mol/L) induced contraction (7.1 ± 1.3% vs. 60 mmol/L KCl) under resting tension, and indomethacin (a cyclooxygenase inhibitor, 10^−5^ mol/L) induced relaxation (3.9 ± 0.9% vs. 10^−4^ mol/L SNP) under a contraction with L-NNA.

### 3.3. Effects of Endothelial Denudation and Cimetidine on Histamine-Induced Contraction

**Figure 3:**
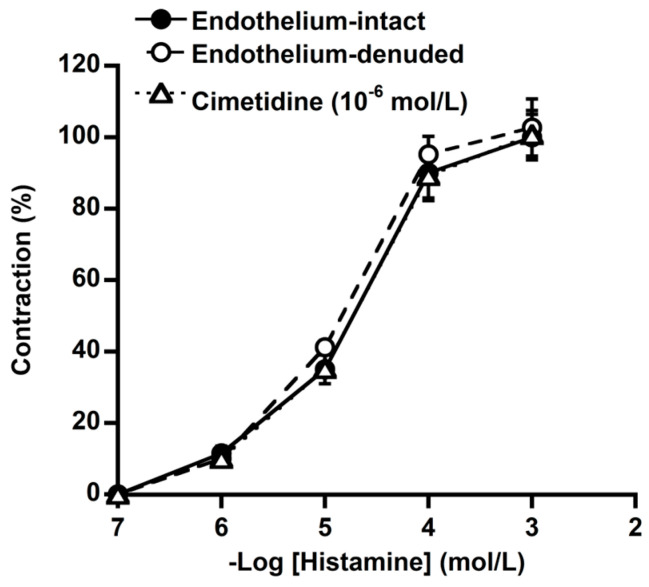
The concentration–response curves of histamine in endothelium-intact (●) and denuded (○) basilar arteries and the effect of cimetidine (△; 10^−6^ mol/L) on histamine-induced contraction (●) in the endothelium-intact basilar arteries of deer. Cimetidine had no effects on the vascular resting tension or the histamine-induced contraction. The contractions induced by 60 mmol/L KCl were considered 100%. Each point represents the mean ± SEM of five deer.

No significant difference was noticed in the histamine-induced contraction between the basilar arteries with intact endothelium and basilar arteries denuded of endothelium (Figure 3). Cimetidine (a H_2_ receptor antagonist, 10^−6^ mol/L) had no significant effect on the histamine concentration–response curve (Figure 3).

### 3.4. Effects of Diphenhydramine on Histamine-Induced Contraction

**Figure 4:**
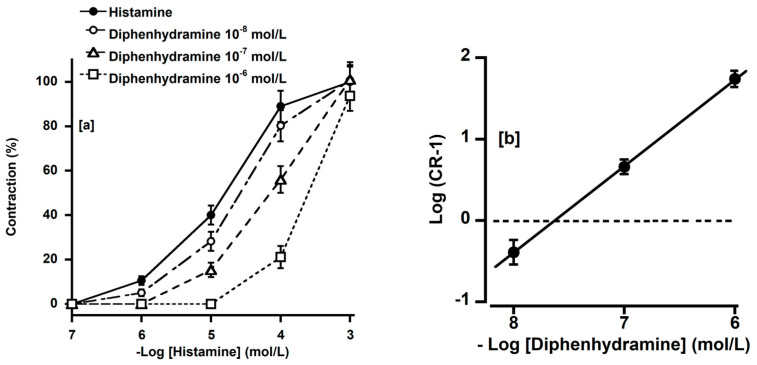
Effects of diphenhydramine (○, 10^−8^; △, 10^−7^; □, 10^−6^ mol/L) on histamine-induced contractions (●) (**a**) and the Schild plot for diphenhydramine (**b**) in the basilar artery of deer. Contractions induced by 10^−3^ mol/L histamine in the absence of diphenhydramine were considered 100%. Diphenhydramine competitively inhibited histamine-induced contraction. Values are expressed as mean ± SEM of five deer. CR, the ratio of equally effective histamine concentrations [50% maximal concentration (EC_50_) in the presence of diphenhydramine/EC_50_ in the absence of diphenhydramine].

We investigated the effects of diphenhydramine (an H_1_ receptor antagonist, 10^−8^–10^−6^ mol/L) on the histamine concentration–response curve. Diphenhydramine shifted the histamine concentration–response curve to the right in parallel (Figure 4a). The calculated pA_2_ value for diphenhydramine was 7.65 ± 0.13, and its slope was 0.89 ± 0.09 (Figure 4b), which did not significantly diverge from unity.

### 3.5. Effect of Endothelial Denudation, L-NNA and Atropine on ACh-Induced Relaxation

**Figure 5:**
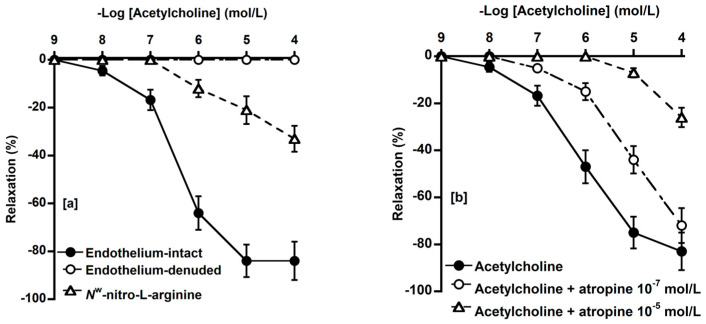
Concentration–response curves of acetylcholine in endothelium-intact (●) and denuded (○) basilar arteries and effect of L-NNA (△, 10^−4^ mol/L) on acetylcholine-induced relaxation (●) (**a**). L-NNA inhibited acetylcholine-induced relaxation. Effect of atropine (○, 10^−7^ mol/L; △, 10^−5^ mol/L) on acetylcholine-induced relaxation (●) in isolated basilar artery of deer (**b**). Atropine competitively inhibited acetylcholine-induced relaxation. U-46619 (10^−7^ mol/L) was used for precontraction. Relaxation induced by 10^−4^ mol/L sodium nitroprusside was considered 100%. Each point represents mean ± SEM for five deer.

We investigated the effects of endothelial denudation, and pretreatment with L-NNA, and treatment with atropine (a nonselective muscarinic receptor antagonist) on the ACh concentration–response curve. The relaxation induced by ACh was completely abolished by endothelial denudation and the curve was significantly shifted rightwards by pretreatment with L-NNA (10^−4^ mol/L) (Figure 5a). Atropine (10^−7^ mol/L and 10^−5^ mol/L) also shifted the ACh concentration–response curve to the right (Figure 5b).

### 3.6. Effect of Pirenzepine, Methoctramine, and pFHHSiD on ACh-Induced Relaxation

**Figure 6:**
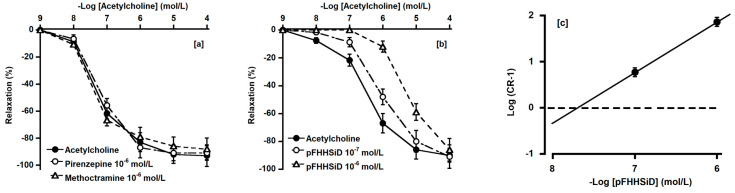
Effect of pirenzepine (○, 10^−6^ mol/L), methoctramine (△, 10^−6^ mol/L) (**a**), and pFHHSiD (○: 10^−7^ mol/L, △: 10^−6^ mol/L) (**b**) on acetylcholine induced relaxation (●) and Schild plot of pFHHSiD (**c**) for basilar artery of deer. U-46619 (10^−7^ mol/L) were used for precontraction. Relaxation induced by 10^−4^ mol/L sodium nitroprusside was considered 100%. Each point represents mean ± SEM for five deer. CR, equally effective ratio of ACh concentrations (EC_50_ in presence of pFHHSiD/EC_50_ in absence of pFHHSiD).

Figure 6a shows the effects of pirenzepine (an M_1_ receptor antagonist) and methoctramine (an M_2_ receptor antagonist) on ACh-induced relaxation during contractions induced by U-46619 (10^−7^ mol/L). Pirenzepine or methoctramine had no significant effect on ACh-induced relaxation. Figure 6b,c shows the effect of pFHHSiD (an M_3_ receptor antagonist) on ACh-induced relaxation under a contraction induced by U-46619 and the relevant Schild plot. pFHHSiD shifted the ACh concentration–response curve to the right. The calculated pA_2_ for pFHHSiD was 7.88 ± 0.16, and its slope was 0.97 ± 0.22 (Figure 6b), which did not significantly diverge from unity.

### 3.7. Relationship Between NA-Induced Response and Gravity

**Figure 7:**
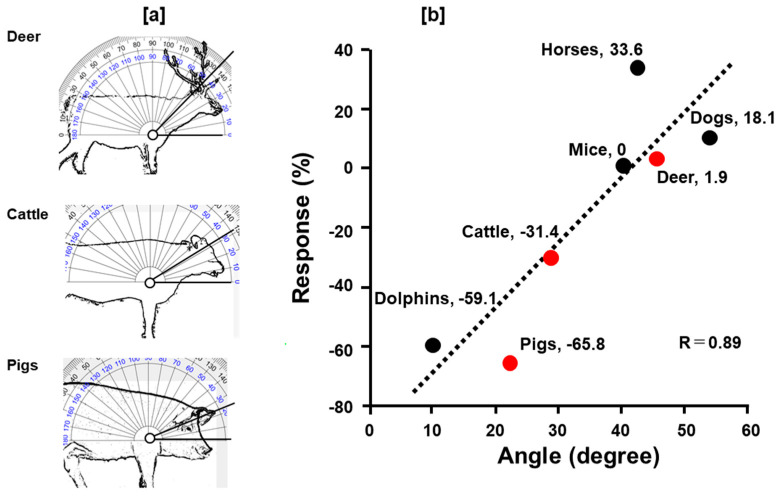
(**a**) Representative examples of the vertical displacement (angle) of the cardiocranial axis from the dorsal plane in even-toed ungulates (deer, cattle and pigs). (**b**) Relationship between the response to noradrenaline and the vertical displacement in mammalian species (dolphins, pigs, cattle, mice, horses, dogs, and deer, n = 3 per species). Data for animals other than dogs and cattle were obtained using the same experimental methods.

To investigate the effects of gravity on adrenergic responses, we measured the vertical displacement (angle) of the cardiocranial axis from the dorsal plane in even-toed ungulate: pigs, cattle and deer (Figure 7a), and dolphins, mice, dog and horses. The data for dolphins [15], pigs [27], mice [9], and horses [17] have been obtained from our group. The data for dogs [31] and cattle [26] were obtained from a previous study by other researchers. The relationship between NA-induced responses and the angle (degree) is graphically shown in Figure 7b. The correlation coefficient was 0.89, indicating a strong positive correlation and significance (*p* < 0.05).

## 4. Discussion

To the best of the authors’ knowledge, this is the first report on basilar arterial vascular responses in a cervid species, the sika deer. We investigated their responses to NA, histamine, ACh, 5-HT, Ang II, and BK, and the receptor subtype populations in the basilar arteries isolated from sika deer carcasses. Our data support the hypothesis that the vertical displacement of the cardiocranial axis from the dorsal plane influences NA-induced responses.

In the basilar arterial rings obtained from slaughtered sika deer, L-NNA and indomethacin induced contraction and relaxation, respectively. Accordingly, we consider that the balance of resting vascular tone may be maintained by the spontaneous release of nitric oxide (NO) and thromboxane A_2_ from endothelial cells, which is a common characteristic of mammalian basilar arteries. Similar results have been obtained in pigs and cattle [22], and horses [32]. Histamine induced concentration-dependent contraction in the cervine basilar arterial rings, and this effect was not affected by endothelial denudation. Histamine induces the endothelium-dependent relaxation of the basilar artery in monkeys by activating H_1_ receptors on endothelial cells and H_2_ receptors on smooth muscle cells [13]. Our results suggest that endothelial cells may not possess histamine receptors and are not involved in the histamine-induced contraction of the basilar artery in deer. Cimetidine did not affect histamine-induced contraction. Diphenhydramine shifted the histamine concentration–response curve to the right. The pA_2_ value for histamine in the cervine basilar arteries in this study (7.65 ± 0.13) was similar to those previously reported in the bovine basilar arteries (7.61 ± 0.11; [11]). These results suggest that H_1_ receptors are present in the smooth muscle cells of the basilar arteries of deer, and their stimulation leads to contraction. Thus, deer may have H_1_ receptors located on smooth muscle cells in their basilar artery, similarly to cattle, but lack the number of H_2_ receptors seen in this artery in cattle [11].

NA induced very weak contractions (1.9% of 60 mmol/L KCl) in the isolated cervine basilar arterial rings in this study. This result differed from the corresponding figures in other even-toed ungulate species, cattle [26] and pigs [27], but were similar to the values reported in dogs and horses, which had a similar vertical displacement of the cardiocranial axis from the dorsal plane. Accordingly, we suggest that this vertical displacement (based on an angle representative of the animal’s normal posture) may affect NA-induced responses in basilar arteries. However, the very weak contraction we noted may be explained by the abolition of the vertical displacement of the cardiocranial axis during prolonged grazing. Further studies evaluating the relationship between the extent of vertical axis displacement and the vasoreactivity elicited by NA are necessary.

Similarly to NA, 5-HT induced minimal contraction (1.2% vs. 60 mmol/L KCl) in this study. We regard this as a highly noteworthy finding because 5-HT induces contractions in most animal species with an intensity ranging between 40 and 100% [33]. 5-HT induces contractions of the bovine anterior cerebral artery, middle cerebral artery, posterior cerebral artery, and basilar artery, all of which are regarded as being more sensitive to 5-HT than the basilar artery [34]. 5-HT induces relaxation of the canine basilar artery [35] and porcine coronary artery [36]. We have previously reported that 5-HT induces weak contraction in the basilar artery of mice in 9 of 36 cases [9].

Ang II and BK did not induce vasomotive action in the cervine basilar arterial rings in this study. Ang II induced a very weak contraction in the porcine cerebral artery, with variations in the response from the proximal to distal parts and variation in the repeated application responses [23]. There are few reports on the response of the bovine basilar artery to Ang II. BK has no vasomotor effects in the bovine basilar artery [22], which is consistent with our finding here in deer; however, it induced endothelium-dependent relaxation followed by contraction in pigs [16] and endothelium-independent contraction in horses [32] and bats [12]. This phenomenon could be explained by species differences or, more specifically, by a distinct cervine kallikrein-kininogen system, which could generate structurally different kinins with different bioactivities from those seen in other species.

In the present study, ACh-induced relaxation was completely abolished by endothelial denudation and was significantly inhibited by L-NNA. These results suggest that ACh induces endothelium-dependent and NO-mediated relaxation in the basilar artery of deer. This contrasts with the bovine basilar artery, where ACh shows no vasomotor effects [16,19]. Atropine shifted the ACh concentration–response curve to the right, thereby suggesting the presence of cholinergic receptors in the cervid basilar artery. Three types of muscarinic receptors are involved in the relaxation or contraction of arteries; however, in the present study, pirenzepine and methoctramine (selective M_1_ and M_2_ receptor antagonists, respectively) had no significant effect, but pFHHSiD (a selective M_3_ receptor antagonist) significantly shifted the ACh concentration–response curve to the right in parallel. The calculated pA_2_ value of 7.88 ± 0.16 for pFHHSiD was similar to that reported for the rat aortas (7.67), where the response was mediated via activation of the M_3_ receptor [37]. Accordingly, we suggest that the M_3_ receptor is involved in ACh-induced relaxation.

In this study on the relationship between noradrenaline responses and vertical displacement, we used mammalian animal models, particularly even-toed ungulates. We anticipate expansion of the scope of future research to include birds, amphibians and reptiles, and further clarification of the causes of the variation between animal species. In the next step in our research, we will clarify the effects of microgravity on the autoregulation of endothelial cells.

## 5. Conclusions

Histamine, NA, and 5-HT induced contraction, whereas ACh induced relaxation, in the cervine basilar artery. Our results indicate that the cerebrovascular responses of deer to histamine, BK, L-NNA, and indomethacin are similar to those seen in cattle, but the responses to NA and ACh are different from those in pigs and cattle. Our findings suggest that cerebrovascular responses reflect similarities in animal species and are influenced by the head and heart position relative to gravity.

## Figures and Tables

**Table 1:** The pEC_50_ values and maximal response (Max) to agonists.

Agonists	pEC_50_	Max (%)
Histamine	5.55 ± 0.06	101.8 ± 2.3 ^a^
Noradrenaline	–	1.9 ± 0.3 ^a^
5-Hydroxytriptamine	–	1.2 ± 0.4 ^a^
Angiotensin II	–	No response
Bradykinin	–	No response
Acetylcholine	7.05 ± 0.01	−88.9 ± 8.3 ^b^

^a^ Contraction induced by 60 mmol/L KCl was taken as 100%. ^b^ Relaxation induced by sodium nitroprusside (10^−4^ mol/L) was taken as 100%. Each point represents the mean ± SEM of five deer.

## Data Availability

All the data included in this study are available, on request, from the corresponding author.
